# A feasibility study to evaluate early treatment response of brain metastases one week after stereotactic radiosurgery using perfusion weighted imaging

**DOI:** 10.1371/journal.pone.0241835

**Published:** 2020-11-03

**Authors:** Jiayi Huang, Mikhail Milchenko, Yuan J. Rao, Pamela LaMontagne, Christopher Abraham, Clifford G. Robinson, Yi Huang, Joshua S. Shimony, Keith M. Rich, Tammie Benzinger

**Affiliations:** 1 Department of Radiation Oncology, Washington University School of Medicine, Saint Louis, Missouri, United States of America; 2 Mallinckrodt Institute of Radiology, Washington University School of Medicine, Saint Louis, Missouri, United States of America; 3 Department of Neurosurgery, Washington University School of Medicine, Saint Louis, Missouri, United States of America; University of Texas MD Anderson Cancer Center, UNITED STATES

## Abstract

**Background:**

To explore if early perfusion-weighted magnetic resonance imaging (PWI) may be a promising imaging biomarker to predict local recurrence (LR) of brain metastases after stereotactic radiosurgery (SRS).

**Methods:**

This is a prospective pilot study of adult brain metastasis patients who were treated with SRS and imaged with PWI before and 1 week later. Relative cerebral blood volume (rCBV) parameter maps were calculated by normalizing to the mean value of the contralateral white matter on PWI. Cox regression was conducted to explore factors associated with time to LR, with Bonferroni adjusted p<0.0006 for multiple testing correction. LR rates were estimated with the Kaplan-Meier method and compared using the log-rank test.

**Results:**

Twenty-three patients were enrolled from 2013 through 2016, with 22 evaluable lesions from 16 patients. After a median follow-up of 13.1 months (range: 3.0–53.7), 5 lesions (21%) developed LR after a median of 3.4 months (range: 2.3–5.7). On univariable analysis, larger tumor volume (HR 1.48, 95% CI 1.02–2.15, p = 0.04), lower SRS dose (HR 0.45, 95% CI 0.21–0.97, p = 0.04), and higher rCBV at week 1 (HR 1.07, 95% CI 1.003–1.14, p = 0.04) had borderline association with shorter time to LR. Tumors >2.0cm^3^ had significantly higher LR than if ≤2.0cm^3^: 54% vs 0% at 1 year, respectively, p = 0.008. A future study to confirm the association of early PWI and LR of the high-risk cohort of lesions >2.0cm^3^ is estimated to require 258 patients.

**Conclusions:**

PWI at week 1 after SRS may have borderline association with LR. Tumors <2.0cm^3^ have low risk of LR after SRS and may be low-yield for predictive biomarker studies. Information regarding sample size and potential challenges for future imaging biomarker studies may be gleaned from this pilot study.

## Introduction

Brain metastases occur in approximately 10–35% of adult cancer patients [1]. For patients with a limited number of brain metastases, stereotactic radiosurgery (SRS) rather than whole brain radiation therapy (WBRT) has increasingly become the preferred treatment [1, 2]. Four randomized trials have collectively demonstrated that SRS alone has superior neurocognitive profile and quality of life than SRS plus WBRT without jeopardizing overall survival (OS) [3–7]. However, SRS alone is associated with worse local recurrence (LR) as well as out-of-field distant brain failure (DBF) than SRS plus WBRT. Notably, among the randomized trials, the one-year LR rates ranged around 27–33% for SRS alone vs 0–11% for SRS plus WBRT [3–5]. Recently, novel RT technique such as hippocampal-avoidance intensity-modulated WBRT (HA-WBRT) has shown comparable disease control while reducing the neurocognitive profile of WBRT [8]. Early biomarkers of tumor response to SRS, ideally within the first weeks after treatment, may aid in identifying high-risk patients who could benefit from additional HA-WBRT or other local salvage therapies such as laser interstitial thermal therapy (LITT) or staged SRS boost [9, 10].

Dynamic susceptibility contrast-enhanced perfusion-weighted magnetic resonance imaging (PWI) has emerged as a promising imaging biomarker for treatment response monitoring for glioblastoma and brain metastasis [11–13]. PWI measures hemodynamic changes that reflect the underlying microvasculature and angiogenesis [14]. Different measurement parameters of PWI have been shown to correlate with tumor microvasculature density, capillary blood volume, and microvasculature leakage [15–17]. Since tumor microvasculature is an essential component of the microenvironment that promotes brain tumor progression and treatment resistance, hemodynamic changes after treatment may reflect tumor response [18, 19].

We previously conducted a preclinical study that showed the dynamic measurement of perfusion changes using positron emission tomography (PET) could differentiate between early radiation necrosis vs viable tumor for tumor xenografts after a single fraction of 15 Gy [20]. Detailed comparison of dynamic PET changes with corresponding histological changes also suggested that the optimal time to differentiate the radioresistant versus radiosensitive region would be 7 days after a single-fraction of high-dose radiotherapy [20]. Similar to dynamic PET, PWI measures perfusion changes within the tumor microenvironment, so we hypothesize that the changes of brain metastases on PWI after SRS can predict LR. Thus, a pilot study was designed to perform PWI on brain metastases before SRS and 1 week later to correlate to local control. The primary objective of this pilot study was to explore the feasibility of whether PWI of brain metastasis 1 week after SRS would be a promising imaging biomarker for LR and to generate preliminary data to inform future studies. The secondary objective was to explore the optimal parameter to analyze PWI for brain metastasis after SRS.

## Materials and methods

### Study design and patients

**Fig 1:**
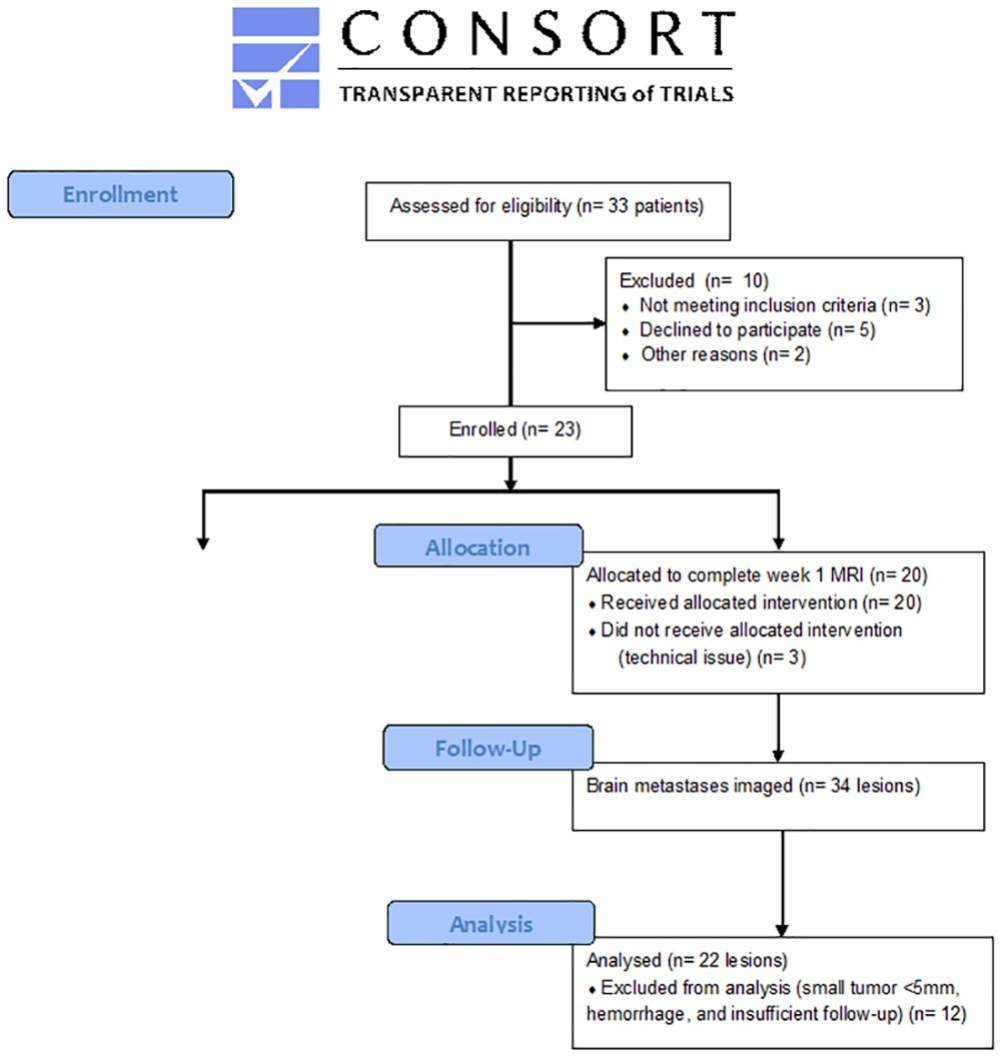
CONSORT flow diagram.

This prospective pilot study was designed to perform PWIs before and 1 week after treatment for newly diagnosed adult brain metastasis patients treated with SRS alone at Washington University in St.Louis/Siteman Cancer Center/Barnes Jewish Hospital (Fig 1). Eligible patients were required to have at least one brain metastasis ≥1cm, age ≥18, Karnofsky Performance Status (KPS) ≥60, and estimated glomerular filtrate rate ≥60 mL/min/1.73m^2^. Patients who had prior brain metastases treated with SRS or WBRT were excluded. After two patients with melanoma were enrolled, their week1 MRIs showed significant tumor hemorrhage that interfered with the processing of perfusion maps. The protocol was subsequently amended to exclude patients with melanoma or hemorrhagic lesions. The protocol was approved by the Washington University in St. Louis Institutional Review Board on 3/5/2013 and was conducted in accordance with the Declaration of Helsinki and Good Clinical Practice guidelines. Patient recruitment was from 7/1/2013 until 6/30/2016, and the last follow-up was ended on 12/31/2018. All patients provided written informed consent before enrollment. The study was registered with ClinicaTrials.gov (NCT02311556) on 12/8/2014. The study was not registered after the initial approval as it did not meet the criteria for mandatory registration, but our institution subsequently changed policy to register all non-therapeutic studies as many journals increasingly require registration regardless of the mandatory criteria.

### SRS

All patients were treated with single-fraction SRS using a Gamma Knife unit (Elekta Medical Systems, Stockholm, Sweden) at Washington University in St. Louis/Siteman Cancer Center. For treatment planning, both a high-resolution contrast-enhanced MRI as well as a non-contrast-enhanced head computed tomography (CT) scan were obtained, and target delineation and treatment planning were completed by a team of radiation oncologist, neurosurgeon, and medical physicist. Prescription doses were based on Radiation Therapy Oncology Group (RTOG) 90–05, and the doses were generally 20–21 Gy for tumors <2 cm in size, 17–18 Gy for tumors 2–3 cm in size, and 15–16 Gy for tumors >3 cm in size. The prescription dose was selected so that the 50% isodose line would cover the gross tumor volume (GTV). After SRS, patients were followed by both radiation oncology and neurosurgery with standard of care serial brain magnetic resonance imaging (MRI) every three months.

### MRI and PWI

MRIs with PWI sequences were performed before SRS and 1 week after SRS (day 7–10). PWI was acquired as dynamic susceptibility-weighted contrast images with tracer method in which bolus injection of gadolinium contrast agent (MultiHance, Bracco Diagnostics Inc., Princeton, NJ; 0.1 mmol/kg) was followed by repeated T2*-weighted gradient echo-planar image acquisition using clinical 12 channel head coils with image resolution of 2.2 x 2.2 mm in plane resolution and spacing between slices of 6.22±0.5 mm, repetition time of 2.2±0.7 s, and echo time of 37±8 ms. PWI sequences were also included as part of the routine MRIs at the week 12 follow-up whenever possible. All MRIs were performed on Siemens scanners (Siemens, Erlangen, Germany). MRIs at week 1 were performed on dedicated 3-Tesla research scanners, MRIs at baseline and week 12 were typically performed on 1.5-Tesla scanners as they were done as part of clinical care.

### Image processing

**Fig 2:**
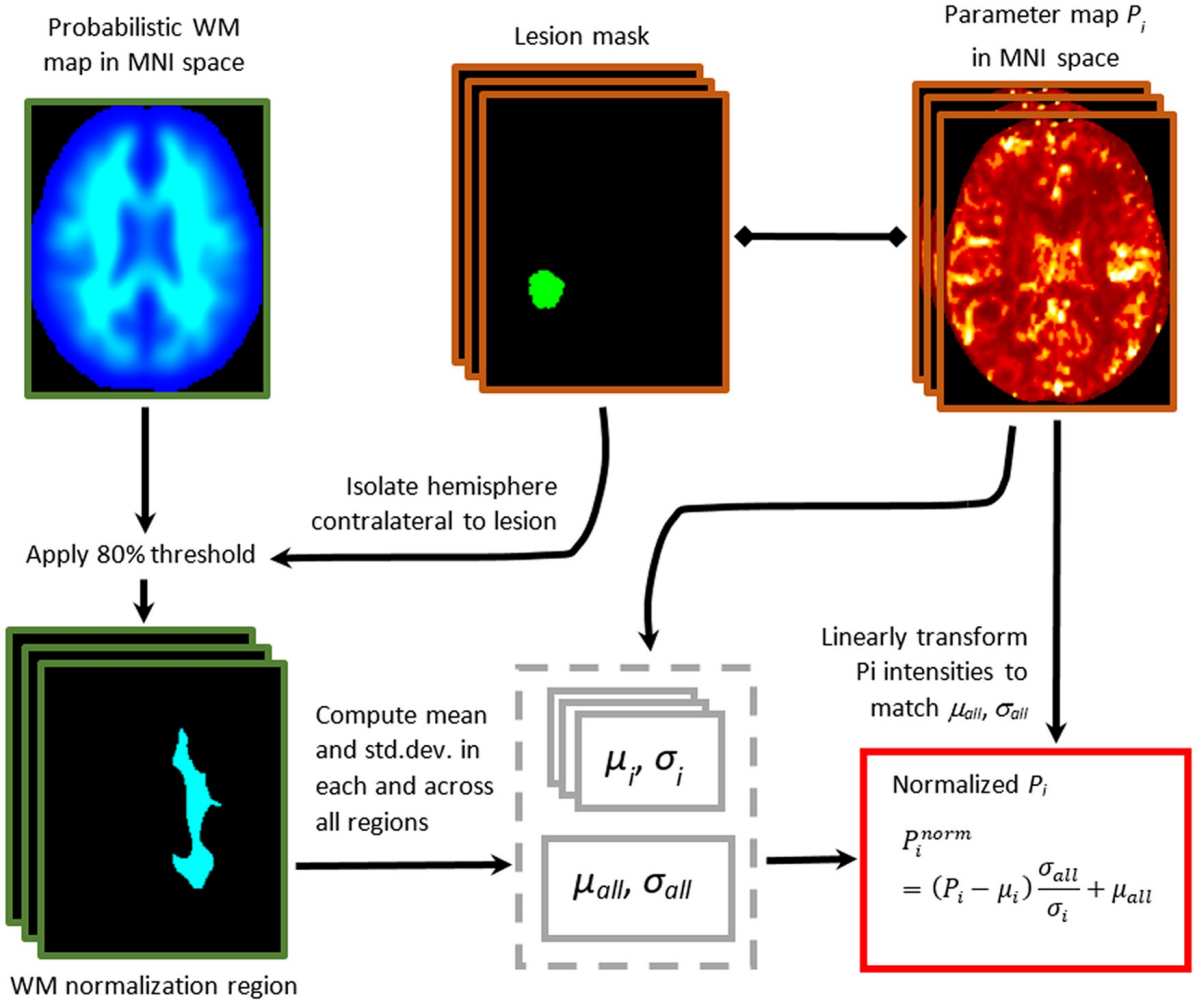
The white matter (WM) normalization process. MNI or MNI152 atlas represents the T1-weighted MRI template derived from 152 healthy adults. P_i_ represents the parameter map of CBV or CBF of the index perfusion scan. μ_i_ and σ_i_ represent the mean and standard deviation of the index parameter map within the corresponding WM normalization region, respectively. μ_all_ and σ_all_ represent the mean and standard deviation computed across all parameter maps in the study, respectively.

All MRIs for each participant were spatially aligned, and perfusion maps were generated using the Multimodal Glioma Analysis pipeline [21]. As a small hand-drawn region of interest (ROI) in an arbitrary region of the contralateral brain may not be representative of the overall white matter (WM), we elected to use the mean value of the contralateral WM for normalization. The probabilistic WM maps, available for MNI152 atlas in FSL 5.0 [22], were binarized for each hemisphere, with voxels of WM probability less than 0.8 set to 0. Regions containing brain metastases in contralateral WM were excluded from these maps. For each CBV and CBF map, the mean and standard deviation of the contralateral WM were computed. Each individual map was then linearly transformed so that the mean and standard deviation would match across all subjects. Since CBV and CBF maps have no fixed units, this procedure was performed to standardize PWI intensity to allow a more accurate comparison. The WM normalization process is demonstrated in Fig 2. The relative CBV (rCBV) and relative CBF (rCBF) values were then computed as the 99^th^, 95^th^, and 50^th^ percentiles within a given lesion ROI normalized to the contralateral WM mean.

### Endpoints

LR was centrally reviewed based on all available clinical, radiologic, and pathologic data and determined according to the updated Response Evaluation Criteria In Solid Tumors (RECIST) 1.1 criteria [23]. LR required ≥20% diameter increase of the treated lesion as compared with the diameter of the nadir after SRS, with a minimum of 5 mm increase over the nadir. Pseudoprogression or radiation necrosis, where an irradiated lesion demonstrated increased size followed by spontaneous stabilization or regression on subsequent MRIs without any tumor-directed therapy, were not considered as LR. Surgical confirmation, when available, was also used to distinguish radiation necrosis from LR. Response assessment was censored at the time of salvage WBRT. An evaluable lesion was defined as an unresected lesion ≥5 mm with at least 3 months of follow-up for response assessment.

### Statistical analysis

As this study is a pilot study, the sample size was empirically set at 20 patients who would complete PWIs at baseline and 1 week after SRS, as such sample size has been shown to provide a reasonable precision for estimating preliminary information to design larger confirmatory studies [24]. Frequency distribution between lesions with or without LR were compared using the Wilcoxon rank-sum test for continuous variables and Fisher’s exact test for categorical variables. The Cox proportional hazards regression method was used to explore factors that were associated with time to LR. Of note, the Cox method was used as a guide to explore different variables for developing future hypothesis and was not used to develop a model to predict LR. Bonferroni adjusted P value <0.006 (0.05/8 tests) was applied for controlling type I error due to multiple tests in the univariable analysis. Multivariable analysis was not performed due to small sample size and limited events. Hoeffding’s D statistic was used to check for correlation between variables. LR rates were estimated with the Kaplan-Meier method and compared using the log-rank test. All time-to-event data were calculated from the date of SRS. All tests were 2-sided. Statistical analyses were performed with the Statistical Package for Social Sciences, version 23.0 (IBM SPSS Statistics, Armonk, NY).

## Results

**Fig 3:**
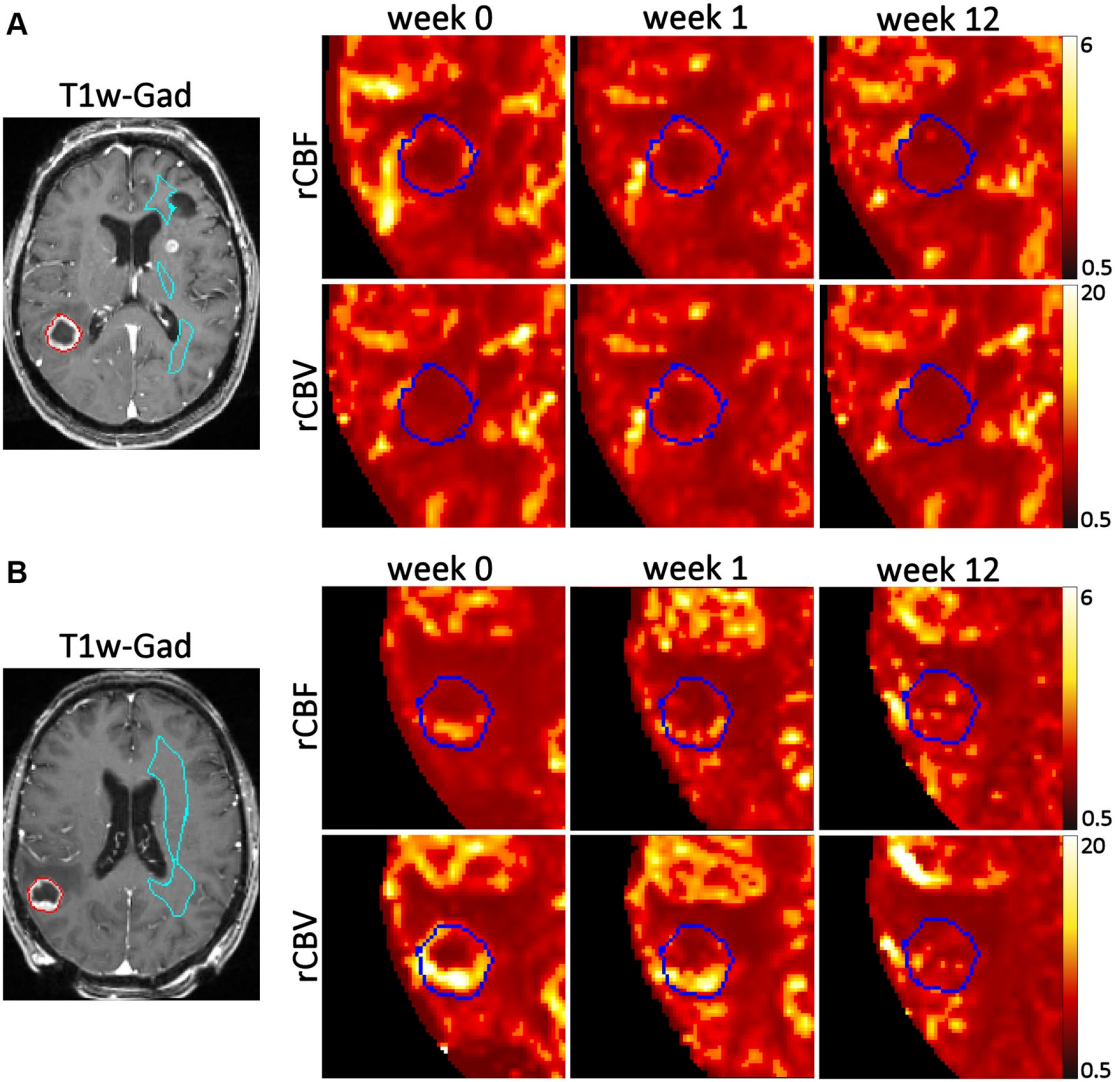
Representative axial MRI and rCBF and rCBV maps before and after radiosurgery. A) A lesion that did not develop local recurrence. B) A lesion that developed local recurrence. Red/dark blue outlines the treated lesion, cyan blue outlines the contralateral white matter normalization region. T1w-Gad represents post-contrast T1-weighed MRI sequence before radiosurgery.

Twenty-three patients were enrolled from 2013 through 2016, with 20 completing the required baseline and week 1 PWIs (Fig 1). Enrollment was slow due to a few reasons: exclusion of patients with prior WBRT or SRS, exclusion of hemorrhagic lesions or melanoma histology, reluctance of patients to undergo additional MRI shortly after SRS, and non-therapeutic nature of the study. The 20 patients had 34 lesions treated, but 12 were not evaluable due to small size with lesion diameter <5 mm (n = 5), tumor hemorrhage at week 1 (n = 2, melanoma histology), and lack of follow-up (n = 5, including 2 lesions with melanoma and renal cell carcinoma that also had tumor hemorrhage at week 1). Lesions <5 mm and hemorrhagic lesions were excluded because they could not be reliably processed for perfusion maps due to image resolution and artifacts from the blood product, respectively. Thus, the final dataset included a total of 22 evaluable lesions (65% of imaged lesions) from 16 patients (70% of enrolled patients). The most common histology was non-small cell lung cancer (10 lesions or 46%), followed by gynecological malignancies (7 lesions from ovarian cancer and 1 lesion from endometrial cancer or 36%). The remaining histologies included colorectal (9%), tonsillar (5%), and thyroid (5%). The characteristics of the patients and their evaluable lesions are described in Table 1. After a median follow-up of 13.1 months (range: 3.0–53.7), five LRs occurred after a median of 3.4 months (range: 2.3–5.7). All five LRs occurred in patients with a single brain metastasis, so there were no discordant responses of different lesions within the same patient. LRs were confirmed with subsequent MRIs (n = 3) or surgical evaluation (n = 1), and the remaining patient enrolled in hospice before the confirmatory MRI. Representative MRIs with corresponding rCBF and rCBV maps before and after SRS for lesions who did or did not develop LR are shown in Fig 3. Qualitatively, the lesions that did not develop LR generally had homogenously low rCBV and rCBF maps at baseline and at week 1, whereas the lesions that developed LR typically showed regional high rCBV at baseline and at week 1. The lesions that developed LR were more likely to have non-gynecological histologies, larger GTV (and diameter), lower SRS dose, as well as higher rCBV and rCBF at week 1 (Table 1). For clarity, only rCBV and rCBF from the 95^th^ percentiles (rCBV95% and rCBF95%, respectively) are presented, and the full data of PWI parameters from the 99^th^ and 50^th^ percentiles can be seen in the S1 Table.

**Table 1:** Patient and lesion characteristics.

Patient characteristics^a^	All evaluable patients (n = 16)	Patients without LR (n = 11)	Patients with LR (n = 5)	P-value^b^
Age	57 (33–73)	55 (40–73)	55 (33–73)	0.91
Sex				0.11
Male	7 (44%)	3 (27%)	4 (80%)	
Female	9 (56%)	8 (73%)	1 (20%)	
KPS	90 (70–100)	90 (70–100)	80 (70–100)	0.52
Lesion characteristics	All evaluable lesions (n = 22)	Lesions without LR (n = 17)	Lesions with LR (n = 5)	P-value
Histology				0.01
NSCLC	10 (46%)	8 (47%)	2 (40%)	
GYN^c^	8 (36%)	8 (47%)	0	
Other^c^	4 (18%)	1 (6%)	3 (60%)	
Tumor diameter (cm)	1.8 (0.5–2.6)	1.6 (0.5–2.6)	2.2 (2.0–2.3)	0.03
GTV (cm^3^)	2.06 (0.10–7.43)	1.51 (0.10–7.43)	4.63 (3.82–6.79)	0.02
SRS dose (Gy)	20 (17–21)	20 (17–21)	18 (17–20)	0.03
Wk0 rCBV95%	6.31 (-0.44–23.27)	6.00 (1.70–22.41)	16.09 (-0.44–23.27)	0.37
Wk0 rCBF95%	1.49 (-0.003–8.33)	1.46 (0.54–8.33)	1.93 (-0.003–5.64)	0.91
Wk1 rCBV95%	6.53 (0.53–40.76)	6.25 (0.53–40.76)	14.63 (5.93–37.80)	0.11
Wk1 rCBF95%	1.41 (0.16–6.48)	1.24 (0.16–6.48)	2.17 (1.40–3.85)	0.046

^a^Continuous variables are presented as median (range), and categorical variables are presented as number (percentages).

^b^P-value was determined using the Wilcoxon rank-sum test for continuous variables and Fisher’s exact test for categorical variables.

^c^Gynecological malignancies included 7 lesions from ovarian cancer and 1 lesion from endometrial cancer; other (histologies) include: 2 colorectal, 1 tonsillar, and 1 thyroid cancer. Abbreviations: KPS = Karnofsky Performance Status, NSCLC = non-small cell lung cancer, GYN = gynecological malignancies, GTV = gross tumor volume, SRS = stereotactic radiosurgery, Wk = week, rCBV = relative cerebral blood volume of tumor as compared to the mean of contralateral white matter, rCBF = relative cerebral blood flow as compared to the mean of contralateral white matter, rCBV95% = rCBV calculated using the 95^th^ percentile of CBV values within the tumor region of interest, LR = local recurrence.

**Fig 4:**
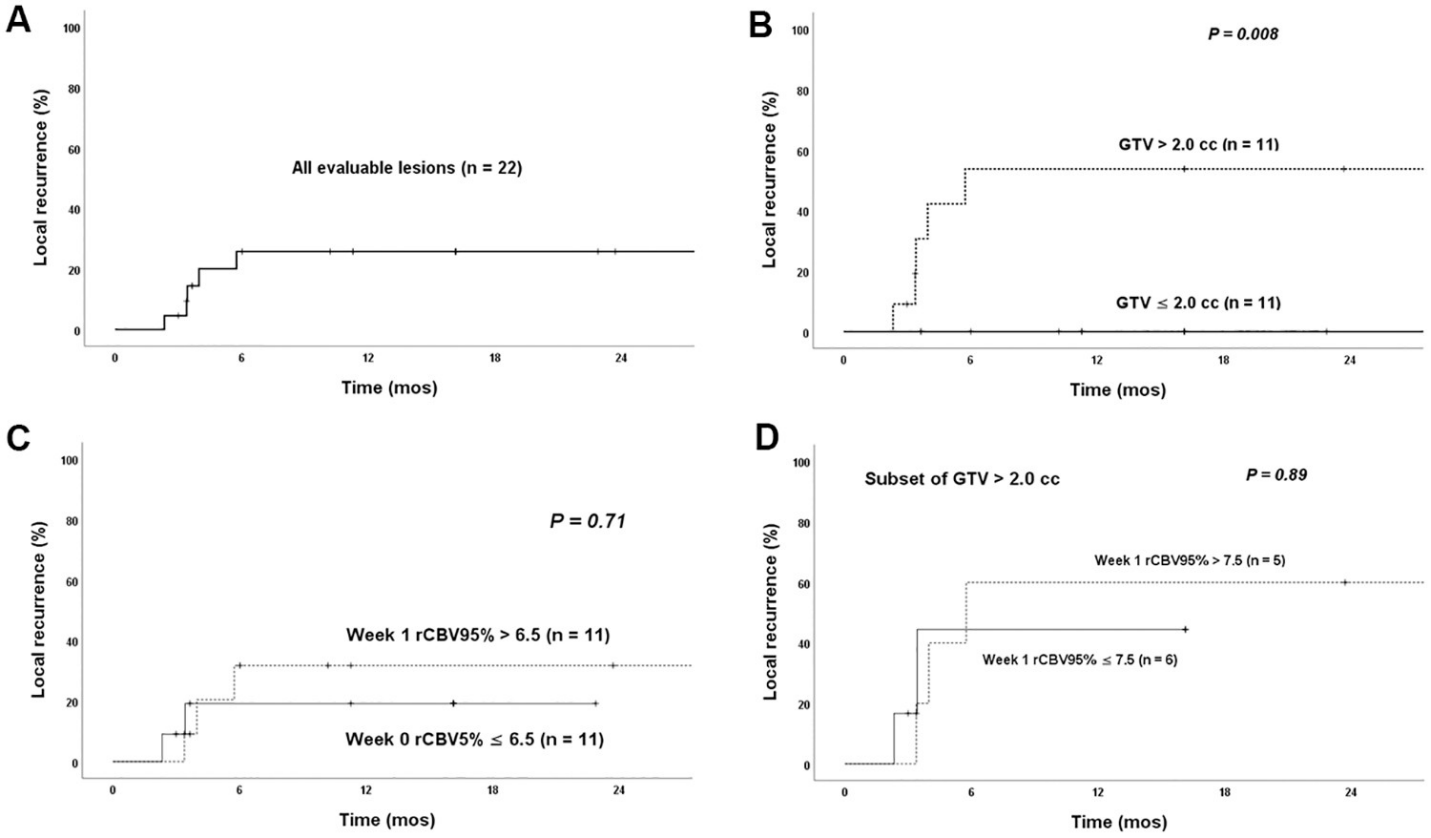
Effect of tumor volume and week 1 perfusion parameter on local recurrence of brain metastasis after radiosurgery. A) Overall Local recurrence rate. B) Local recurrence rate stratified by gross tumor volume (GTV). C) Local recurrence rate stratified by the median rCBV95% value at week 1 after SRS. D) Local recurrence rate of the subset of lesions with GTV > 2.0 cc stratified by the median rCBV95% value at week 1 after SRS. rCBV95% = relative cerebral blood volume calculated using the 95th percentile of CBV values within the tumor normalized by the mean of contralateral white matter.

**Fig 5:**
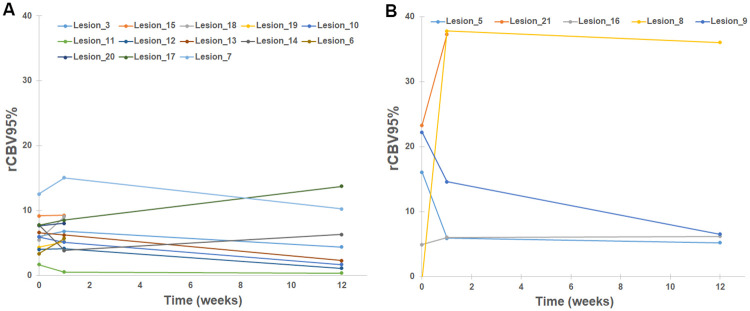
Spaghetti plot of changes of perfusion parameter rCBV over time for lesions that developed local recurrence vs not. A) Lesions that did not develop local recurrence with at least 6 months of follow-up (n = 13, excluding 4 lesions with <6 months of follow-up). B) Lesions that developed local recurrence (n = 5). rCBV95% = relative cerebral blood volume calculated using the 95th percentile of CBV values within the tumor normalized by the mean of contralateral white matter.

Not surprisingly, patient factors such as age, sex, and KPS were not associated with development of LR for individual patients (S2 Table). When evaluating the association of lesion-specific characteristics with time to LR for individual lesions, none of the variables were significant according to the Bonferroni correction. Larger GTV (HR 1.48, 95% CI 1.02–2.15, p = 0.04), lower SRS dose (HR 0.45, 95% CI 0.21–0.97, p = 0.04), and higher week1 rCBV95% (HR 1.07, 95% CI 1.003–1.14, p = 0.04) had borderline association with shorter time to LR, but they were not significant due to small sample size and multiple testing (Table 2). Of note, there was no correlation between tumor volume and rCBV and rCBF parameters. Among different methods to calculate rCBV, rCBV95% appeared to have more robust association with LR than rCBV50% and rCBV99% (S3 Table). The predictive impact of the borderline variables were further evaluated using the Kaplan-Meier method and their median as the cutoff value. The one-year LR rate of the entire cohort was 26% (Fig 4A). GTV >2.0 cm^3^ had significantly higher LR rate than GTV ≤2.0 cm^3^: 54% vs 0% at one year, respectively, p = 0.008 (Fig 4B). Stratification by SRS dose was similar to GTV but less dramatic (not surprising given SRS dose was selected based on GTV): one-year LR of 55% for ≤18 Gy vs 8% for >18 Gy, respectively, p = 0.02. In contrast, higher week1 rCBV95% (>6.5) did not have significantly different LR than lower rCBV95% (≤6.5): 32% vs 19% at one year, respectively, p = 0.71 (Fig 4C). For the subset of 11 tumors with GTV >2.0 cm^3^ (median week 1 rCBV95% = 7.5), higher week 1 rCBV95% (>7.5) was again not associated with significantly different LR than lower rCBV95% (≤7.5): 60% vs 44% at one year, respectively, p = 0.89 (Fig 4D). As seen in Fig 5, rCBV95% of most lesions showed a downward trend in the first three months after SRS. The lesions that developed LR tend to have higher rCBV at baseline and week 1, but there was significant overlap between the two cohorts.

**Table 2:** Univariable analysis of factors associated with local recurrence.

	All evaluable lesions (n = 22)	
	HR (95% CI)	P-value^a^
Histology^b^		0.17
NSCLC	Ref	--
Gyn	N/A^d^	0.97
Other	5.57 (0.91–34.03)	0.06
Larger diameter^c^ (cm)	8.82 (0.95–81.8)	0.06
Larger GTV^c^ (cm^3^)	1.48 (1.02–2.15)	0.04
Higher SRS dose^c^ (Gy)	0.45 (0.21–0.97)	0.04
Wk0 rCBV95%^c^	1.11 (0.99–1.25)	0.07
Wk0 rCBF95%^c^	1.24 (0.84–1.84)	0.28
Wk1 rCBV95%^c^	1.07 (1.003–1.14)	0.04
Wk1 rCBF95%^c^	1.45 (0.88–2.39)	0.15

^a^Determined using the Cox proportional hazards regression method.

^b^Categorical variable.

^c^Continuous variable.

^d^Not applicable to estimate HR due to zero events.

Abbreviations as Table 1.

Given the current study is a pilot study, its main goal is to obtain preliminary information about early PWI as an imaging biomarker to predict LR to help to design future studies. Given our analysis (Table 2) shows that 3 variables (GTV, SRS dose, and week 1 rCBV95%) appear to have borderline association with LR, a sufficiently powered study in the future would need at least 45 events to confirm any association of these 3 variables with LR, as each continuous variable would need at least 15 events to establish a reliable Cox model. If the next study would focus on the high-risk cohort of lesions >2.0 cm^3^ with one year LR of approximately 25% based on historical SRS data [25], then 180 evaluable patients would be needed. Given our study showed that approximately 30% of enrolled patients would not be evaluable, approximately 258 patients would need to be enrolled.

## Discussion

This prospective pilot study identified some obstacles to test PWI as an early imaging predictor of LR of brain metastasis after SRS, including high attrition rate (approximately 30%), patient reluctance of additional imaging in the absence of therapeutic benefit, limited image resolution for tumors smaller than 0.5cm, and inability to image tumors of certain histologies with propensity to hemorrhage such as melanoma or renal cell carcinoma. This study confirms the previous observations that small tumors < 2.0cm^3^ have very low risk of LR after SRS, so they are probably not high-yield candidates for future predictive biomarker studies. Although this small pilot study observed a borderline association between week 1 rCBV95% with LR, a larger study of 258 patients with tumors > 2cm^3^ would be needed for validation.

A few previous studies have supported rCBV as a promising biomarker to predict clinical outcomes after RT [11–13]. Notably, Essig et al prospectively followed 18 brain metastasis patients treated with SRS and correlated the radiological responses at 6 months with their PWIs at baseline, 6 weeks, and 12 weeks after SRS. They showed that the pre-treatment rCBV was not correlated to response, but the relative changes of mean rCBV at week 6 and week 12 were. They calculated the rCBV using the regional mean CBV from the tumor minus the necrotic region and a selected region of 100 pixels in the unaffected brain. They only tested correlation with radiological response at 6 month using the chi-square test and did not check for predictive association with LR which would be the more clinically relevant endpoint [12]. Jakubovic et al has previously conducted a larger prospective study of 44 brain metastasis patients examining rCBV, rCBF, and K^2^_trans_ at week 1 and week 4 after either WBRT or SRS to predict LR and radiological response. K^2^_trans_ was derived from a different type of PWI called dynamic contrast enhancement (DCE) MRI. They showed that lower rCBV at week 1 was predictive of LR but not at week 4. The sensitivity and specificity of rCBV at week 4 for LR was 74% and 82%, respectively. In contrast, lower K^2^_trans_ at week 1 was predictive of radiological response but not LR. Although their study was larger, it was also more heterogeneous and included patients receiving WBRT (23%) as well as those with prior RT (55%) [26]. Their observed association between lower rCBV with recurrent tumors contradicts with prior data that suggest higher rCBV is more likely associated with recurrent tumors [11–13]. The counter-intuitive observation may be due to their method of analysis where the mean of CBV within a ROI was used for calculating rCBV and that they did not exclude necrotic region from determining the mean CBV of the tumor. Altogether, the data appear to suggest that early rCBV (at week 1 or 4 after SRS) may have moderate association with LR, but a study with relatively large sample size would be required for validation.

The discrepancy between PWI processing among different studies highlights a challenge to develop PWI as an accurate imaging biomarker. Since a small area of aggressive disease may drive LR and the necrotic center of a lesion may have relative low perfusion values, evaluation using the median or the mean may not be robust, whereas manual exclusion of the necrotic center is labor intensive and subjective. In contrast, using the maximum or top 99^th^ percentile may be more sensitive to noise. Our study shows that evaluation using the top 95th percentile of CBV values within tumor ROIs is more robust than using the median and maybe slightly better than the top 99th percentile (S3 Table). An arbitrary small ROI in the contralateral brain may not be representative of the overall WM, thus we proposed to use the mean value of the standardized contralateral WM. Lack of standardization of PWI intensity before computing rCBV and rCBF may also introduce variation, so we incorporated a procedure to standardize PWI intensity in our image processing. Larger studies to determine the optimal method for PWI processing may be warranted.

This prospective pilot study is designed to explore the potential of early PWI 1 week after SRS as a biomarker to predict LR. We previously conducted a preclinical study that treated mouse xenografts with a single-fraction of 15 Gy and evaluated tumor perfusion changes using serial dynamic PET imaging. We showed that imaging after 1 week was the optimal time point to differentiate the radioresistant versus radiosensitive regions before tumor regrowth [20]. We subsequently conducted another preclinical study of weekly dynamic PET imaging of mouse xenografts during 5 weeks of fractionated RT and showed perfusion change after 1 week was more predictive of LR than at later time points [27]. This pilot clinical study provides preliminary data to suggest that early perfusion changes of tumor on PWI have moderate association with LR. This study also highlights a limitation of PWI related to tumor hemorrhage, which may limit its application for many tumor histologies such as melanoma or renal cell carcinoma. Novel PET imaging tracers, such as ^18^F-FET or ^18^F-FDOPA, may warrant further investigation as early imaging biomarkers that may overcome the limitations of PWI [28].

Consistent with published literature, this study confirms that GTV is one of the most impactful factors to predict LR of brain metastasis after SRS. Baschnagel et al have analyzed a cohort of 423 brain metastases treated with SRS and showed that GTV >2.0 cm^3^ was associated with significantly worse local control than <2.0 cm^3^: 75% vs 97% at 1 year, respectively, p<0.001 [25]. Other large institutional series have also reported similar results with local control of tumors < 2.0 cm^3^ consistently greater than 95% [29, 30]. Since tumors <2.0 cm^3^ have consistently shown relatively low risk of LR after SRS, future imaging biomarker studies should exclude these small lesions and concentrate on the high-risk cohort of lesions >2.0 cm^3^, which should improve positive predictive accuracy. Since the larger tumors are more likely to recur, there is also a greater need to identify accurate early imaging biomarkers to improve tumor control.

As this study is a relatively small prospective pilot study, its findings should be considered as hypothesis generating for future studies. Due to the limited number of events in this study and correction for multiple testing, none of the variables demonstrates significant association. In particular, rCBV95% on week 1 PWI shows weak discrimination for LR, and an estimated sample size of 258 is required to confirm significant association, which may be financially and logistically difficult to implement, especially considering other limitations of PWI. The pilot study has relatively high proportion of gynecological malignancies, which is not representative of the overall brain metastasis population. However, this should not significantly affect the main study findings. The difference of magnet strength between week1 MRIs versus baseline/week12 MRIs may limit our analysis of serial comparisons. However, a comparative imaging study of a cohort of 30 glioma patients with both 1.5-Tesla and 3-Tesla scanners has also previously reported excellent reproducibility and correlation of rCBV regardless of the magnet strength [31]. Since rCBVs are calculated by normalizing to the contralateral normal brain from each scan, it may be less sensitive to variation from different magnet strength or scanners. Future studies on early imaging biomarkers for brain metastasis may consider evaluation of other novel PET techniques and integration within therapeutic studies.

## Supporting information

S1 Table Detailed perfusion MRI parameters using the 99^th^, 95^th^, and 50^th^ percentiles. (DOCX)

S2 Table Univariable analysis of patient characteristics associated with local recurrence. (DOCX)

S3 Table Univariable analysis of perfusion MRI parameters associated with local recurrence. (DOCX)

S1 File Study protocol. (DOCX)

S2 File TREND checklist. (PDF)

S3 File De-identified dataset. (XLSX)
