# Supplementary material for: A feasibility study to evaluate early treatment response of brain metastases one week after stereotactic radiosurgery using perfusion weighted imaging
Source: PLoS One. 2020 Nov 3;15(11):e0241835. doi: 10.1371/journal.pone.0241835 (PMC7608872; doi:10.1371/journal.pone.0241835)
Supplement: S1 Table — (DOCX) [file pone.0241835.s001.docx]

**S1 Table. Detailed perfusion MRI parameters using the 99^th^, 95^th^, and 50^th^ percentiles**

| Parameters^a^ | All evaluable lesions (n = 22) [median (range)] | Lesions without LR (n = 17)  [median (range)] | Lesions with LR  (n = 5)  [median (range)] | P-value^b^ |
| --- | --- | --- | --- | --- |
| Wk0 rCBV99% | 9.13 (1.01-33.65) | 8.93 (1.77-33.65) | 20.99 (1.01-31.54) | 0.37 |
| Wk0 rCBV95% | 6.31 (-0.44-23.27) | 6.00 (1.70-22.41) | 16.09 (-0.44-23.27) | 0.37 |
| Wk0 rCBV50% | 2.46 (-1.67-10.69) | 2.38 (-0.16-10.69) | 6.12 (-1.67-9.82) | 0.51 |
| Wk0 rCBF99% | 1.91 (0.16-17.16) | 1.91 (0.57-17.16) | 2.55 (0.16-6.68) | 0.97 |
| Wk0 rCBF95% | 1.49 (-0.003-8.33) | 1.46 (0.54-8.33) | 1.93 (-0.003-5.64) | 0.91 |
| Wk0 rCBF50% | 0.53 (-0.25-3.36) | 0.52 (0.16-3.36) | 0.64 (-0.25-2.11) | 0.78 |
| Wk1 rCBV99% | 8.12 (0.70-53.44) | 7.55 (0.70-53.44) | 22.05 (7.73-49.10) | 0.07 |
| Wk1 rCBV95% | 6.53 (0.53-40.76) | 6.25 (0.53-40.76) | 14.63 (5.93-37.80) | 0.11 |
| Wk1 rCBV50% | 1.85 (-1.69-15.80) | 1.76 (-1.69-15.80) | 3.46 (1.55-12.21) | 0.26 |
| Wk1 rCBF99% | 1.92 (0.30-8.44) | 1.83 (0.30-8.44) | 3.12 (1.90-5.33) | 0.04 |
| Wk1 rCBF95% | 1.41 (0.16-6.48) | 1.24 (0.16-6.48) | 2.17 (1.40-3.85) | 0.046 |
| Wk1 rCBF50% | 0.50 (-0.04-2.28) | 0.51 (-0.04-2.28) | 0.46 (0.36-1.72) | 0.67 |

^a^All parameters listed below are continuous variables.

^b^P-value was determined using the Wilcoxon rank-sum test.

Abbreviations: Wk = week, rCBV = relative cerebral blood volume of tumor as compared to the mean of contralateral white matter, rCBF = relative cerebral blood flow as compared to the mean of contralateral white matter, rCBV99% = rCBV calculated using the 99^th^ percentile of CBV values within the tumor region of interest, LR = local recurrence.
