# Supplementary material for: A feasibility study to evaluate early treatment response of brain metastases one week after stereotactic radiosurgery using perfusion weighted imaging
Source: PLoS One. 2020 Nov 3;15(11):e0241835. doi: 10.1371/journal.pone.0241835 (PMC7608872; doi:10.1371/journal.pone.0241835)
Supplement: S2 Table — (DOCX) [file pone.0241835.s002.docx]

**S2 Table. Univariable analysis of patient characteristics associated with local recurrence**

| Variables | All patients (n = 16) | |
| --- | --- | --- |
|  | HR (95% CI) | P-value^a^ |
| Older age^b^ | 0.99 (0.91-1.08) | 0.83 |
| Male sex^c^ | 7.60 (0.82-70.11) | 0.07 |
| Higher KPS^b^ | 0.97 (0.90-1.05) | 0.49 |

^a^Determined using the Cox proportional hazards regression method.

^b^Continuous variable.

^c^Categorical variable.
