# Supplementary material for: A feasibility study to evaluate early treatment response of brain metastases one week after stereotactic radiosurgery using perfusion weighted imaging
Source: PLoS One. 2020 Nov 3;15(11):e0241835. doi: 10.1371/journal.pone.0241835 (PMC7608872; doi:10.1371/journal.pone.0241835)
Supplement: S3 Table — (DOCX) [file pone.0241835.s003.docx]

**S3 Table. Univariable analysis of perfusion MRI parameters associated with local recurrence**

| Parameters^a^ | All evaluable lesions (n = 22) | |
| --- | --- | --- |
|  | HR (95% CI) | P-value^b^ |
| Wk0 rCBV99% | 1.07 (0.99-1.16) | 0.10 |
| Wk0 rCBV95% | 1.11 (0.99-1.25) | 0.07 |
| Wk0 rCBV50% | 1.29 (0.98-1.69) | 0.07 |
| Wk0 rCBF99% | 1.05 (0.83-1.32) | 0.70 |
| Wk0 rCBF95% | 1.24 (0.84-1.84) | 0.28 |
| Wk0 rCBF50% | 1.80 (0.70-4.60) | 0.22 |
| Wk1 rCBV99% | 1.05 (1.001-1.11) | 0.04 |
| Wk1 rCBV95% | 1.07 (1.003-1.14) | 0.04 |
| Wk1 rCBV50% | 1.13 (0.94-1.37) | 0.20 |
| Wk1 rCBF99% | 1.33 (0.89-1.97) | 0.16 |
| Wk1 rCBF95% | 1.45 (0.88-2.39) | 0.15 |
| Wk1 rCBF50% | 1.91 (0.38-9.58) | 0.43 |

^a^All parameters listed below are continuous variables.

^b^Determined using the Cox proportional hazards regression method.

Abbreviations as S1 Table.
