# Supplementary material for: A feasibility study to evaluate early treatment response of brain metastases one week after stereotactic radiosurgery using perfusion weighted imaging
Source: PLoS One. 2020 Nov 3;15(11):e0241835. doi: 10.1371/journal.pone.0241835 (PMC7608872; doi:10.1371/journal.pone.0241835)
Supplement: S1 File — (DOCX) [file pone.0241835.s004.docx]

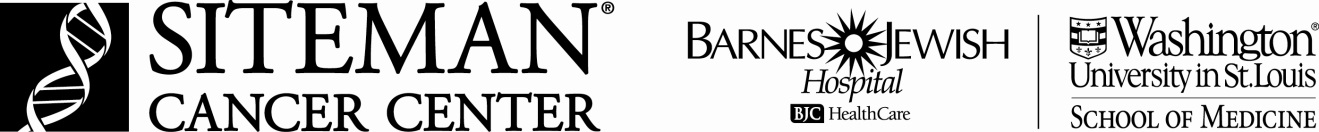


**Assessment of Early Treatment Response of Brain Metastases after Gamma-Knife Radiosurgery Using Dynamic Susceptibility-weighted Contrast-enhanced Perfusion Magnetic Resonance Imaging: A Pilot Study**

**Protocol Version Date: 08/27/14**

**HRPO#: 201302018**

**Principal Investigator: Jiayi Huang, M.D.**

Washington University in St Louis

Department of Radiation Oncology

4921 Parkview Place / Box 8224

St. Louis, MO 63110

Telephone: 314-362-8516

Fax: 314-747-9557

Email: [jhuang@radonc.wustl.edu](mailto:jhuang@radonc.wustl.edu)

**Radiology Co-PI:** Tammie Benzinger, M.D., Ph.D.

Washington University in St Louis

Department of Radiology

660 South Euclid Ave / Box 8131

St. Louis, MO 63110

**Neurosurgery Co-PI:** Keith Rich, M.D.

Washington University in St Louis

Department of Radiology

660 South Euclid Ave / Box 8057

St. Louis, MO 63110

**Sub-Investigators:** **Institution: Department:**

Clifford Robinson, M.D. Washington University Radiation Oncology

Joseph Simpson, M.D., Ph.D. Washington University Radiation Oncology

Albert Kim, M.D., Ph.D. Washington University Neurosurgery

Todd DeWees, Ph.D. Washington University Biostatistics/Rad Onc

**Assessment of Early Treatment Response of Brain Metastases after Gamma-Knife Radiosurgery Using Dynamic Susceptibility-weighted Contrast-enhanced Perfusion Magnetic Resonance Imaging: A Pilot Study**

**Protocol Revision History**

Initial Approval 01/30/13

Amendment #1 07/26/13

Amendment #2 05/13/14

Amendment #3 08/27/14

**TABLE OF CONTENTS**

[SCHEMA 4](#_Toc398792612)

[1 BACKGROUND 5](#_Toc398792613)

[1.1 Preliminary Studies of Dynamic PET for Early Response Monitoring 5](#_Toc398792614)

[1.2 Correlative Studies of DSC-PMR 6](#_Toc398792615)

[1.3 Significance 6](#_Toc398792616)

[1.4 Interim Analysis 6](#_Toc398792617)

[2 OBJECTIVES 7](#_Toc398792618)

[2.1 Primary Aim 7](#_Toc398792619)

[2.2 Secondary Aim 7](#_Toc398792620)

[3 PATIENT ELIGIBILITY 7](#_Toc398792621)

[3.1 Inclusion Criteria 7](#_Toc398792622)

[3.2 Exclusion Criteria 7](#_Toc398792623)

[4 PATIENT REGISTRATION 8](#_Toc398792624)

[4.1 Confirmation of Patient Eligibility 8](#_Toc398792625)

[4.2 Patient Registration in the Siteman Cancer Center Database 8](#_Toc398792626)

[4.3 Assignment of UPN 8](#_Toc398792627)

[5 METHODS 8](#_Toc398792628)

[5.1 Pre-Scan Data Collection 8](#_Toc398792629)

[5.2 MRI and DSC-PMR 9](#_Toc398792630)

[5.3 Data Management 9](#_Toc398792631)

[5.4 Processing of DSC-PMR 10](#_Toc398792632)

[5.5 Follow-Up 10](#_Toc398792633)

[5.6 Evaluation of Suspected Tumor Recurrence 10](#_Toc398792634)

[6 STUDY CALENDAR 11](#_Toc398792635)

[7 DATA SUBMISSION SCHEDULE 11](#_Toc398792636)

[8 REGULATORY AND REPORTING REQUIREMENTS 11](#_Toc398792637)

[8.1 Adverse Events (AEs) 12](#_Toc398792638)

[8.2 Unanticipated Problems 12](#_Toc398792639)

[8.3 Noncompliance 12](#_Toc398792640)

[8.4 Serious Noncompliance 12](#_Toc398792641)

[8.5 Protocol Exceptions 13](#_Toc398792642)

[8.6 Reporting to the Human Research Protection Office (HRPO) and the Quality Assurance and Safety Monitoring Committee (QASMC) at Washington University 13](#_Toc398792643)

[8.7 Timeframe for Reporting Required Events 13](#_Toc398792644)

[9 DATA AND SAFETY MONITORING 14](#_Toc398792645)

[10 STATISTICAL CONSIDERATIONS 14](#_Toc398792646)

[10.1 Sample Size 14](#_Toc398792647)

[10.2 Study Endpoint 14](#_Toc398792648)

[10.3 Definition of Clinical Outcomes 15](#_Toc398792649)

[10.4 Statistical Tests 15](#_Toc398792650)

[11 REFERENCES 16](#_Toc398792651)

[APPENDIX A: Karnofsky Performance Status (KPS) Scale 18](#_Toc398792652)

[APPENDIX B: CLINICAL FOLLOW-UP FORMS 19](#_Toc398792653)

[APPENDIX C: DSC-PMR FORMS 20](#_Toc398792654)

# SCHEMA

Patients with brain metastases elected for

Gamma-knife (GK) alone without

whole brain radiation therapy (WBRT)

Standard GK (with MRI + perfusion MR)

1 week (7-10 days) after GK: MRI + perfusion MR

Routine follow-up with standard MRI every 3 months

# BACKGROUND

Brain metastases occur in 20% to 40% of patients with cancer (1). Traditionally, whole brain radiation therapy (WBRT) has been the standard treatment approach (2). More recently, stereotactic radiosurgery (SRS) has gained popularity in treating those with limited brain metastases (3, 4). SRS is a technique that employs multiple convergent beams of radiation to provide a large, highly conformal dose to a selected target. It is typically delivered using a Cobalt-60-based device (also known as Gamma-Knife (GK)) or a linear accelerator (4). The Siteman Cancer Center at Washington University serves as a major regional referral center for GK and treats over 100 patients with brain metastasis per year.

Recent randomized studies have demonstrated equivalence in survival between those treated with SRS alone versus SRS and WBRT (5, 6). Addition of WBRT was associated with significant decline in learning and memory recall at 4 months after treatment (7). However, omission of WBRT was associated with increased risk of local recurrence as well as elsewhere-brain failure (5-7). The one-year local tumor recurrence rate after SRS alone ranged around 27-33% in the three randomized studies, as contrasted to 0-19% after SRS and WBRT (5-7). Currently, selection between SRS alone versus SRS plus WBRT for treatment of limited brain metastases is controversial (4).

Accurate and non-invasive imaging that can provide early prediction of treatment response to SRS would provide the much needed guidance for the treatment selection. For those with poor response to SRS, additional WBRT may be added to prevent tumor recurrence. This adaptive approach would promote individualized therapy to increase the therapeutic ratio. Furthermore, novel radiotherapy technique such as hippocampal-sparing intensity-modulated WBRT may allow increased dose to the resistant lesions while reducing the neuro-cognitive effect of WBRT by decreasing the dose to the hippocampus (8, 9). However, before the adaptive approach can be incorporated in routine clinical practice, the most crucial first step is to identify an accurate and non-invasive imaging method for early treatment response monitoring.

Advancements in magnetic resonance imaging (MRI) techniques have allowed for non-invasive assessment of physiological changes. Notably, dynamic susceptibility-weighted contrast-enhanced perfusion MRI (DSC-PMR) is a promising imaging technique that may be used as a biomarker for treatment response monitoring. DSC-PMR exploits signal changes that accompany the passage of a gadolinium-based contrast agent through the cerebrovascular system. It measures hemodynamic changes that reflect the underlying microvasculature and angiogenesis (10). Different measurement parameters of DSC-PMR have been shown to correlate with tumor microvasculature density, capillary blood volume, and microvasculature leakage (11-13). Since tumor microvasculature is an important component of the microenvironment that promotes brain tumor progression and treatment resistance (14, 15), its changes after treatment may reflect tumor response.

## Preliminary Studies of Dynamic PET for Early Response Monitoring

Using small animal imaging, we have previously shown that dynamic positron emission tomography (PET) may provide informative assessment of early radiation necrosis and radiation change of tumor xenografts after a single dose of radiotherapy (16). Detailed comparison of PET changes with corresponding histological changes also suggests that the optimal timing to differentiate the radioresistant versus radiosensitive region would be 7 days after a single-fraction of high-dose radiotherapy, with 30 days being the next optimal time point (16). Follow-up studies have shown that PET parameters that measure perfusion changes during the first week of fractionated radiotherapy might be predictive of tumor control. Although PET is not routinely used to measure perfusion changes in brain tumors, the principles demonstrated by the animal studies are thought provoking.

## Correlative Studies of DSC-PMR

Three clinical studies of DSC-PMR have suggested that perfusion changes during or shortly after radiotherapy may correlate to tumor response. In a prospective study of 23 glioma patients treated with conformal radiotherapy, Cao *et al.* conducted DSC-PMR at Weeks 1 and 3 during the radiotherapy. They found that fractional changes of cerebral blood volume (CBV) on DSC-PMR were predictive for survival (17). In another retrospective study of 36 glioma patients treated with radiotherapy and temozolomide, Mangla *et al.* reported that increased magnitude of relative CBV from DSC-PMR at one month after treatment was associated with significantly worse survival (18). The third study performed DSC-PMR on 18 patients with brain metastases at 6 weeks and at 3 months after SRS. The study showed that high regional CBV value at 6 weeks after SRS was predictive of tumor recurrence, with a sensitivity of 91% and a specificity of 71% (19). However, this study only performed DSC-PMR at 6 weeks after SRS and did not image tumors during the first two weeks after SRS.

## Significance

Although there is no direct therapeutic benefit to the participant for participating in this study, the study offers promising potential benefit to society, with important clinical implication on the management of brain metastases. If DSC-PMR can accurately assess for early treatment response after GK as hypothesized, hippocampal-sparing intensity-modulated WBRT may be selectively used to decrease local tumor recurrence for high risk patients while avoiding unnecessary irradiation for low risk patients. This would have an important implication as we continue to push for personalized medicine in cancer treatment. We would expect that the data generated from this pilot study will lead to larger grant to fund studies to incorporate DSC-PMR for a phase II study for incorporating hippocampal-sparing intensity-modulated WBRT after SRS to provide individualized radiation therapy for patients with brain metastases. Furthermore, the additional MRI at Weeks 1 and 4 may provide indirect benefit to the participants by allow early detection of new brain metastases, intracranial bleeding or stroke.

## Interim Analysis

During our interim analysis after enrollment of 11 patients, we determined that compliance for the Week 4 scan has been very poor. It is unlikely that any meaningful data will be obtained by having any additional patients complete the Week 4 scan. The study objectives have been revised to clarify that the analysis of the Week 1 scan is the primary objective and that the analysis of Week 4 scan is the secondary objective. After the approval of this amendment, the Week 4 scan will not be obtained for the remaining patients.

# OBJECTIVES

## Primary Aim

To evaluate and compare changes of tumor perfusion on DSC-PMR occurring between at Week 1 after GK versus at baseline (before GK) as a predictor for time to local tumor progression. Specific quantitative values of relative cerebral blood volume (rCBV), relative cerebral blood flow (rCBF), relative peak height (rPH), and percentage of signal-intensity recovery (PSR) will be determined from each DSC-PMR. The changes of these parameters will be correlated with local control after GK to identify their predictive accuracy.

# PATIENT ELIGIBILITY

## Inclusion Criteria

1. Patient must have newly diagnosed brain metastases visible on brain MRI. A biopsy of the lesion is not required as long as the patient has a biopsy-proven malignancy elsewhere and a clinician deems the lesion to be metastatic.
2. At least one brain metastasis must be ≥ 1cm to allow adequate quantitative imaging measurement for DSC-PMR.
3. Patient must be clinically eligible for and elect to be treated with GK alone without WBRT.
4. Patient must be ≥ 18 years of age.
5. Patient must have Karnofsky Performance Status (KPS) of at least 60 (see Appendix A).
6. Patient may be part of other clinical trials (as long as no other local treatments beyond GK such as WBRT or other local therapy are indicated to the brain) or imaging studies.
7. Patient or legally authorized representative must be able to understand and willing to sign a written informed consent document.

## Exclusion Criteria

1. Patient must not have any medical contraindications to MRI (e.g., unsafe foreign metallic implants, incompatible pacemaker, inability to lie still for long periods, severe to end-stage kidney disease or on hemodialysis).
2. Patient must not be pregnant or breastfeeding.
3. Patient must not have an estimated glomerular filtration rate (eGFR) < 60 mL/min/1.73m^2^.
4. Patient must not have melanoma.
5. Patient must not have hemorrhagic lesions.

# PATIENT REGISTRATION

Patients must not start any protocol intervention prior to registration through the Siteman Cancer Center. Patients may be consented and registered either before or after Gamma Knife surgery.

The following steps must be taken before registering patients to this study:

1. Confirmation of patient eligibility
2. Registration of the patient in the Siteman Cancer Center database
3. Assignment of unique patient number (UPN)

## Confirmation of Patient Eligibility

Confirmation of patient eligibility includes the information listed below:

1. Registering coordinator’s name and contact information
2. Registering MD’s name
3. Patient’s race, sex, and DOB
4. Copy of signed consent form
5. Completed eligibility checklist, signed and dated by a member of the study team
6. Copy of appropriate source documentation confirming patient eligibility

## Patient Registration in the Siteman Cancer Center Database

Patients must be registered through the Siteman Cancer Center database.

## Assignment of UPN

Each patient will be identified with a unique patient number (UPN) for this study. Patients will also be identified by first, middle, and last initials. If the patient has no middle initial, a dash will be used on the case report forms (CRFs). All data will be recorded with this identification number on the appropriate CRFs.

# METHODS

## Pre-Scan Data Collection

Data from a standard of care pre-treatment H&P (including KPS) and pre-treatment MRI of the brain will be collected in the CRFs.

Data from the standard radiosurgery treatment for brain metastasis will be collected in the CRFs, including number and location of lesions treated. As per routine care, patients will undergo standard MRI and CT for tumor localization and treatment planning. Data from this imaging will be collected in the CRFs as well. Prior to GK, patients must have undergone DSC-PMR as part of one pre-treatment scan. DSC-PMR is routinely included in the standard MRI on the day of GK as part of our routine clinical practice. If it has been performed as part of the standard pre-treatment MRI, it is not required at the time of GK. All pre-treatment procedures and evaluations, and treatment with GK itself, is part of routine care and is not dictated by this protocol; data from these evaluations will be collected in the case report forms as part of the on-study data.

## MRI and DSC-PMR

Additional MRIs with DSC-PMR will be performed at Week 1 (range: 7-10 days) after GK. Given that this additional MRI scan is not routine clinical practice, it will be performed at 3 Tesla combined PET-MRI scanner in the Center for Clinical Imaging Research (CCIR) for logistic reasons. The MRI component of the CCIR PET-MRI scanner is identical to the 3 Tesla clinical MRI scanner; PET imaging will not be utilized during this study. In the event the CCIR PET-MRI scanner is down, then clinical MRI scanner will be used. The standard FDA-approved head coil will be used. DSC-PMR will be performed with tracer method in which gadolinium contrast agents is injected and subsequent T2*-weighted gradient echo-planar images are acquired as the contrast passes through the brain. The DSC-PMR scanning time is approximately 3-5 minutes. The MRI scan at Weeks 1 will also include standard sequences such as T1 with and without contrast, so the total scanning time will be 30 minutes.

All participants scheduled to undergo gadolinium contrast enhanced MRI will be evaluated for potential renal insufficiency by calculation of an estimated glomerular filtration rate (eGFR) using the standard calculator provided by the NIH/National Kidney Disease Education Program (found at <http://www.nkdep.nih.gov/professionals/gfr_calculators/orig_con.htm>).  Plasma creatinine (mg/dL) shall be obtained from the medical record, or if none is available from the medical record within the last 90 days, it will be obtained on the day of scanning, prior to MRI.  If the contrast is administered for clinical purposes, the standard Barnes Jewish Hospital/Mallinckrodt Institute of Radiology algorithm for contrast dosing shall be used.  Contrast shall not be administered for research purposes in participants with an eGFR < 60 mL/min/1.73m^2^.

## Data Management

Clinical MR images will be sent to the Clinical Desktop system and will have standard clinical interpretations provided by the Department of Radiology. The investigational MRI imaging at Week 1 after GK will be reviewed by Dr. Tammie Benzinger (co-principal investigator) and reported in the Clinical Desktop system. This represents potential benefit to the patients, as incidental findings of new brain metastases, intracranial bleeding, or stroke may be detected early. For clinical sequences, post-processing will be performed according to standard protocols. In addition, research and clinical MR data without identifying patient information will be archived on the Central Neuroimaging Data Archive (CNDA) to allow for long-term follow up.

## Processing of DSC-PMR

All MRI data will undergo coregistration and will be resampled into atlas space for viewing and processing. DSC-PMR will be processed to obtain rCBV, rCBF, rPH, PSR for each region of interest (22), see Appendix C. Regions of interest (ROI) will be generated based upon the clinical T1-post contrast and FLAIR images. ROI will include the enhancing tumor volume, the non-enhancing area of T2 hyperintensity surrounding the enhancing tumor, and a matching ROI from normal brain in the contralateral hemisphere. Given the DSC-PMR data will not be used in clinical care, the parameters in Appendix C may be completed in batch up to 6 months from the completion of radiosurgery.

## Follow-Up

Standard clinical follow up will occur every 3 months from the date of GK for at least 12 months as per standard of care. Standard MRI imaging is routinely done prior to each follow-up visit. Of note, at Week 1 after GK, protocol patients will only have the MRI with DSC-PMR without a clinical visit. Specific information regarding local tumor recurrence, complete response, elsewhere-brain failure, clinical deterioration (in the form of KPS score), radiation necrosis, and overall survival should be assessed at each visit and will be audited by research coordinators periodically. Follow-up beyond this time will not be tracked for this study and will be at the discretion of the treating physician.

## Evaluation of Suspected Tumor Recurrence

If local recurrence or progression is detected or suspected on follow-up MRIs in the region of prior GK, routine work-up with serial MRI, DSC-PMR, or PET scans may be done at the discretion of the treating physician. Surgical resection or biopsy for confirmation should be considered if clinically feasible; however, this is not mandated by the protocol and will be at the discretion of the treating physician.

# STUDY CALENDAR

|  | **Baseline ^D^** | **Week 1** | **Follow-Up ^A^** |
| --- | --- | --- | --- |
| Physical exam, medical history, KPS | X |  | X |
| MRI w/contrast | X |  | X |
| Blood for plasma creatinine | X |  | X |
| GK | X |  |  |
| DSC-PMR | X ^C^ | X |  |
| Adverse event assessment | X ^B^ | X ^B^ |  |

A: Standard follow-up will be done at every 3 months after GK for at least one year as part of routine clinical practice. The research coordinator will audit the medical record to record outcomes as documented during routine follow-up.

B: Patients will be followed for adverse events for 8 hours following each research DSC-PMR scan. If the baseline scan is performed as part of the patient’s routine care, patients need not be monitored for AEs as that procedure is performed outside the scope of this protocol.

C: At least one baseline DSC-PMR must be done, either at screening or time of GK (or both).

D: Data will be collected from the indicated standard of care pre-treatment assessments, but these assessments are not dictated by the protocol.

# DATA SUBMISSION SCHEDULE

| **Case Report Form** | **Time point** |
| --- | --- |
| Consent Form | At time of consent |
| Eligibility Checklist  Registration  On-Study Form | At time of registration |
| GK Form | At time of GK |
| DSC-PMR Form | Baseline/screening or at time of GK (or both)  1 week after GK  [*The DSC-PMR parameters in Appendix C May be completed up to 6 months from the time of radiosurgery] |
| Adverse Events Form | AE information will be collected during the 8-hour period following each research DSC-PMR scan |
| Follow-Up Form | 3 months after GK  6 months after GK  9 months after GK  12 months after GK |

# REGULATORY AND REPORTING REQUIREMENTS

Since this is a diagnostic study that does not involve any experimental forms of cancer therapy, adverse event reporting is expected to be minimal. Any medical treatment needed for an adverse reaction to the MRI would be taken care of in the clinic or consultation room by the investigators or by the patient’s regular physician. If any adverse events occur, they will be graded according to CTCAE version 4.

Participants will be monitored for adverse events during the actual imaging period. Participants who experience an adverse event up to 8 hours post-scan will be instructed to inform the treating physician or principal investigator, who will then complete the appropriate reporting forms.

## Adverse Events (AEs)

Definition: any unfavorable medical occurrence in a human subject including any abnormal sign, symptom, or disease.

Grading: the descriptions and grading scales found in the revised NCI Common Terminology Criteria for Adverse Events (CTCAE) version 4.0 will be utilized for all toxicity reporting. A copy of the CTCAE version 4.0 can be downloaded from the CTEP website.

Attribution (relatedness), Expectedness, and Seriousness: the definitions for the terms listed that should be used are those provided by the Department of Health and Human Services’ Office for Human Research Protections (OHRP). A copy of this guidance can be found on OHRP’s website:

<http://www.hhs.gov/ohrp/policy/advevntguid.html>

## Unanticipated Problems

Definition:

- unexpected (in terms of nature, severity, or frequency) given (a) the research procedures that are described in the protocol-related documents, such as the IRB-approved research protocol and informed consent document; and (b) the characteristics of the subject population being studied;
- related or possibly related to participation in the research (in this guidance document, possibly related means there is a reasonable possibility that the incident, experience, or outcome may have been caused by the procedures involved in the research); and
- suggests that the research places subjects or others at a greater risk of harm (including physical, psychological, economic, or social harm) than was previously known or recognized.

## Noncompliance

Definition: failure to follow any applicable regulation or institutional policies that govern human subjects research or failure to follow the determinations of the IRB.  Noncompliance may occur due to lack of knowledge or due to deliberate choice to ignore regulations, institutional policies, or determinations of the IRB.

## Serious Noncompliance

Definition: noncompliance that materially increases risks, that results in substantial harm to subjects or others, or that materially compromises the rights or welfare of participants.

## Protocol Exceptions

Definition: A planned deviation from the approved protocol that are under the research team’s control. Exceptions apply only to a single participant or a singular situation.

Pre-approval of all protocol exceptions must be obtained prior to the event.

## Reporting to the Human Research Protection Office (HRPO) and the Quality Assurance and Safety Monitoring Committee (QASMC) at Washington University

The PI is required to promptly notify the IRB of the following events:

- Any unanticipated problems involving risks to participants or others which occur at WU, any BJH or SLCH institution, or that impacts participants or the conduct of the study.
- Noncompliance with federal regulations or the requirements or determinations of the IRB.
- Receipt of new information that may impact the willingness of participants to participate or continue participation in the research study.

These events must be reported to the IRB within **10 working days** of the occurrence of the event or notification to the PI of the event.  The death of a research participant that qualifies as a reportable event should be reported within **1 working day** of the occurrence of the event or notification to the PI of the event.

## Timeframe for Reporting Required Events

Reportable adverse events will be tracked for 8 hours following each research MRI.

| Deaths | |
| --- | --- |
| Any reportable death while on study or within 30 days of study | Immediately, within 24 hours, to PI and the IRB |
| Any reportable death while off study | Immediately, within 24 hours, to PI and the IRB |
| Adverse Events/Unanticipated Problems | |
| Any reportable adverse events as described in Sections 8.1 and 8.2 (other than death) | Immediately, within 24 hours to PI and within 10 working days to the IRB |
| All adverse events regardless of grade and attribution should be submitted cumulatively | Include in DSM report |
| Noncompliance and Serious Noncompliance | |
| All noncompliance and serious noncompliance as described in Sections 8.3 and 8.4 | Immediately, within 24 hours, to PI and within 10 working days to the IRB |

# DATA AND SAFETY MONITORING

In compliance with the Washington University Institutional Data and Safety Monitoring Plan, the Principal Investigator will provide a Data and Safety Monitoring (DSM) report to the Washington University Quality Assurance and Safety Monitoring Committee (QASMC) semi-annually beginning six months after accrual has opened (if at least five patients have been enrolled) or one year after accrual has opened (if fewer than five patients have been enrolled at the six-month mark). This report will include:

- HRPO protocol number, protocol title, Principal Investigator name, data coordinator name, regulatory coordinator name, and statistician
- Date of initial HRPO approval, date of most recent consent HRPO approval/revision, date of HRPO expiration, date of most recent QA audit, study status, and phase of study
- History of study including summary of substantive amendments; summary of accrual suspensions including start/stop dates and reason; and summary of protocol exceptions, error, or breach of confidentiality including start/stop dates and reason
- Study-wide target accrual and study-wide actual accrual
- Protocol activation date
- Average rate of accrual observed in year 1, year 2, and subsequent years
- Expected accrual end date
- Objectives of protocol with supporting data and list the number of participants who have met each objective
- Early stopping rules with supporting data and list the number of participants who have met the early stopping rules
- Summary of toxicities
- Abstract submissions/publications
- Summary of any recent literature that may affect the safety or ethics of the study

The study principal investigator and study coordinator will monitor for serious toxicities on an ongoing basis. Once the principal investigator or study coordinator becomes aware of a reportable adverse event (RAE), the RAE will be reported to the HRPO and QASM Committee according to institutional guidelines.

# STATISTICAL CONSIDERATIONS

## Sample Size

This is a nonrandomized imaging study with a pre-specified sample size of 20 patients. Different metastatic lesions treated with radiosurgery will be analyzed independently. Based on institutional experience, the average number of lesions is estimated at 3 per patient giving us a sample size of approximately 60 for the primary endpoint of local control.

## Study Endpoint

The primary endpoint of the study is time to local progression of each treated and evaluable brain metastasis. Secondary endpoints will include radiation necrosis, complete response, elsewhere-brain failure, and clinical deterioration.

## Definition of Clinical Outcomes

Local tumor recurrence versus radiation necrosis can be a difficult diagnostic dilemma. The gold standard for this diagnosis will be based upon pathological diagnosis, when available. We would recommend any suspicious lesion be investigated and biopsied. If biopsy is not possible, the patient will be followed clinically and radiographically. Cases where clinical and radiographic findings demonstrate progressive disease, as defined by the MacDonald criteria (20), will be considered as recurrent tumor. Radiation necrosis will be defined by pathology, when available. When no pathology is available, we will use the final clinical diagnosis based on clinical course and management. Complete response is defined as no residue enhancing lesion on MRI. Elsewhere brain failure is defined as appearance of brain metastases outside of prior GK region, which can be diagnosed radiographically and does not require pathological confirmation. Clinical deterioration will be assessed based on the KPS scale (Appendix A).

## Statistical Tests

As it is not certain whether the absolute values of DSC-PMR parameters after treatment or the relative change of those parameters from the baseline scan would provide the best predictive value, we will analyze our DSC-PMR data using both methods. Time to local recurrence will be modeled using the Cox proportional hazard models. The Kaplan-Meier product-limit method will be used to estimate local control probabilities. Logistic repeated measures analysis of variance will be used to model the results of MR perfusion scans from baseline to Week 1 with respect to local progression. Receiver operating characteristic (ROC) curve and area under the ROC curve will be used to estimate the predictive accuracy of different DSC-PMR parameters in predicting local recurrence after GK.

# REFERENCES

1. Patchell RA. The management of brain metastases. *Cancer Treat Rev* 2003;29:533-540.

2. Gelber RD, Larson M, Borgelt BB*, et al.* Equivalence of radiation schedules for the palliative treatment of brain metastases in patients with favorable prognosis. *Cancer* 1981;48:1749-1753.

3. Muller-Riemenschneider F, Bockelbrink A, Ernst I*, et al.* Stereotactic radiosurgery for the treatment of brain metastases. *Radiother Oncol* 2009;91:67-74.

4. Suh JH. Stereotactic radiosurgery for the management of brain metastases. *N Engl J Med* 2010;362:1119-1127.

5. Aoyama H, Shirato H, Tago M*, et al.* Stereotactic radiosurgery plus whole-brain radiation therapy vs stereotactic radiosurgery alone for treatment of brain metastases: a randomized controlled trial. *JAMA* 2006;295:2483-2491.

6. Kocher M, Soffietti R, Abacioglu U*, et al.* Adjuvant whole-brain radiotherapy versus observation after radiosurgery or surgical resection of one to three cerebral metastases: results of the EORTC 22952-26001 study. *J Clin Oncol* 2011;29:134-141.

7. Chang EL, Wefel JS, Hess KR*, et al.* Neurocognition in patients with brain metastases treated with radiosurgery or radiosurgery plus whole-brain irradiation: a randomised controlled trial. *Lancet Oncol* 2009;10:1037-1044.

8. Gondi V, Tolakanahalli R, Mehta MP*, et al.* Hippocampal-sparing whole-brain radiotherapy: a "how-to" technique using helical tomotherapy and linear accelerator-based intensity-modulated radiotherapy. *Int J Radiat Oncol Biol Phys* 2010;78:1244-1252.

9. Hsu F, Carolan H, Nichol A*, et al.* Whole brain radiotherapy with hippocampal avoidance and simultaneous integrated boost for 1-3 brain metastases: a feasibility study using volumetric modulated arc therapy. *Int J Radiat Oncol Biol Phys* 2010;76:1480-1485.

10. Cha S, Knopp EA, Johnson G*, et al.* Intracranial mass lesions: dynamic contrast-enhanced susceptibility-weighted echo-planar perfusion MR imaging. *Radiology* 2002;223:11-29.

11. Cha S, Lupo JM, Chen MH*, et al.* Differentiation of glioblastoma multiforme and single brain metastasis by peak height and percentage of signal intensity recovery derived from dynamic susceptibility-weighted contrast-enhanced perfusion MR imaging. *AJNR Am J Neuroradiol* 2007;28:1078-1084.

12. Law M, Cha S, Knopp EA*, et al.* High-grade gliomas and solitary metastases: differentiation by using perfusion and proton spectroscopic MR imaging. *Radiology* 2002;222:715-721.

13. Lupo JM, Cha S, Chang SM*, et al.* Dynamic susceptibility-weighted perfusion imaging of high-grade gliomas: characterization of spatial heterogeneity. *AJNR Am J Neuroradiol* 2005;26:1446-1454.

14. Charles N, Holland EC. The perivascular niche microenvironment in brain tumor progression. *Cell Cycle* 2010;9:3012-3021.

15. Tredan O, Galmarini CM, Patel K*, et al.* Drug resistance and the solid tumor microenvironment. *J Natl Cancer Inst* 2007;99:1441-1454.

16. Huang J, Chunta JL, Amin M*, et al.* Detailed Characterization of the Early Response of Head-Neck Cancer Xenografts to Irradiation Using (18)F-FDG-PET Imaging. *Int J Radiat Oncol Biol Phys* 84(2):485-91, 2012.

17. Cao Y, Tsien CI, Nagesh V*, et al.* Survival prediction in high-grade gliomas by MRI perfusion before and during early stage of RT [corrected]. *Int J Radiat Oncol Biol Phys* 2006;64:876-885.

18. Mangla R, Singh G, Ziegelitz D*, et al.* Changes in relative cerebral blood volume 1 month after radiation-temozolomide therapy can help predict overall survival in patients with glioblastoma. *Radiology* 2010;256:575-584.

19. Essig M, Waschkies M, Wenz F*, et al.* Assessment of brain metastases with dynamic susceptibility-weighted contrast-enhanced MR imaging: initial results. *Radiology* 2003;228:193-199.

20. Macdonald DR, Cascino TL, Schold SC, Jr.*, et al.* Response criteria for phase II studies of supratentorial malignant glioma. *J Clin Oncol* 1990;8:1277-1280.

# APPENDIX A: Karnofsky Performance Status (KPS) Scale

| **Scale** | **Description** |
| --- | --- |
| 100 | Normal no complaints; no evidence of disease. |
| 90 | Able to carry on normal activity; minor signs or symptoms of disease. |
| 80 | Normal activity with effort; some signs or symptoms of disease. |
| 70 | Cares for self; unable to carry on normal activity or to do active work. |
| 60 | Requires occasional assistance, but is able to care for most of his personal needs. |
| 50 | Requires considerable assistance and frequent medical care. |
| 40 | Disabled; requires special care and assistance. |
| 30 | Severely disabled; hospital admission is indicated although death not imminent. |
| 20 | Very sick; hospital admission necessary; active supportive treatment necessary. |
| 10 | Moribund; fatal processes progressing rapidly. |
| 0 | Dead |

# APPENDIX B: CLINICAL FOLLOW-UP FORMS

| Name of Patient: |  |
| --- | --- |
| Date of Birth: |  |
| Date of GK: |  |
| Date of Visit: |  |
| KPS Score: | (This is used to track clinical deterioration.) |
| Date of recent MRI: |  |
| Elsewhere Brain Recurrence (Outside GK) |  |
| *If yes, treatment: |  |

Local Tumor Response after Initial GK:

|  | Tumor Location | Complete Response* | Local Failure* | Radiation Necrosis* |
| --- | --- | --- | --- | --- |
| 1 |  |  |  |  |
| 2 |  |  |  |  |
| 3 |  |  |  |  |
| 4 |  |  |  |  |
| 5 |  |  |  |  |
| 6 |  |  |  |  |
| 7 |  |  |  |  |
| 8 |  |  |  |  |
| 9 |  |  |  |  |
| 10 |  |  |  |  |
| 11 |  |  |  |  |
| 12 |  |  |  |  |
| 13 |  |  |  |  |
| 14 |  |  |  |  |
| 15 |  |  |  |  |

*Please only write “X” if positive for the corresponding event, ie if tumor completely disappeared after GK, write “X” under “Complete response”. Leave the column blank if the corresponding event has not occurred.

COMMENTS:

# APPENDIX C: DSC-PMR FORMS

| Name of Patient: |  |
| --- | --- |
| Date of Birth: |  |
| Date of GK: |  |
| DSC-PMR #  (1=screening/baseline  2= date of GK  3= week 1 |  |
| Date of DSC-PMR |  |

|  | Tumor Location | rCBV* | rCBF* |
| --- | --- | --- | --- |
| 1 |  |  |  |
| 2 |  |  |  |
| 3 |  |  |  |
| 4 |  |  |  |
| 5 |  |  |  |
| 6 |  |  |  |
| 7 |  |  |  |
| 8 |  |  |  |
| 9 |  |  |  |
| 10 |  |  |  |
| 11 |  |  |  |
| 12 |  |  |  |
| 13 |  |  |  |
| 14 |  |  |  |
| 15 |  |  |  |

*rCBV = relative cerebral blood volume, rCBF = relative cerebral blood flow, rPH = relative peak height, PSR = percentage of peak-signal recovery.

COMMENTS:
